# Notch and Presenilin Regulate Cellular Expansion and Cytokine Secretion but Cannot Instruct Th1/Th2 Fate Acquisition

**DOI:** 10.1371/journal.pone.0002823

**Published:** 2008-07-30

**Authors:** Chin-Tong Ong, John R. Sedy, Kenneth M. Murphy, Raphael Kopan

**Affiliations:** 1 Department of Developmental Biology, Washington University School of Medicine, Saint Louis, Missouri, United States of America; 2 Department of Pathology and Center for Immunology, Washington University School of Medicine, St. Louis, Missouri, United States of America; 3 Howard Hughes Medical Institute, Washington University School of Medicine, St. Louis, Missouri, United States of America; 4 Division of Dermatology, Department of Medicine, Washington University School of Medicine, Saint Louis, Missouri, United States of America; New York University School of Medicine, United States of America

## Abstract

Recent reports suggested that Delta1, 4 and Jagged1, 2 possessed the ability to instruct CD4^+^ T cell into selection of Th1 or Th2 fates, respectively, although the underlying mechanism endowing the cleaved Notch receptor with memory of ligand involved in its activation remains elusive. To examine this, we prepared artificial antigen-presenting cells expressing either DLL1 or Jag1. Although both ligands were efficient in inducing Notch2 cleavage and activation in CD4^+^ T or reporter cells, the presence of Lunatic Fringe in CD4^+^ T cells inhibited Jag1 activation of Notch1 receptor. Neither ligand could induce Th1 or Th2 fate choice independently of cytokines or redirect cytokine-driven Th1 or Th2 development. Instead, we find that Notch ligands only augment cytokine production during T cell differentiation in the presence of polarizing IL-12 and IL-4. Moreover, the differentiation choices of naïve CD4^+^ T cells lacking γ-secretase, RBP-J, or both in response to polarizing cytokines revealed that neither presenilin proteins nor RBP-J were required for cytokine-induced Th1/Th2 fate selection. However, presenilins facilitate cellular proliferation and cytokine secretion in an RBP-J (and thus, Notch) independent manner. The controversies surrounding the role of Notch and presenilins in Th1/Th2 polarization may reflect their role as genetic modifiers of T-helper cells differentiation.

## Introduction

Naive CD4^+^ T cells can acquire at least four distinct phenotypes following activation by antigen, including three distinct types of effectors, Th1, Th2 and Th17 cells and various subsets of regulatory T cells [1], [2], [3], [4], [5], [6], [7]. Among the factors that influence the choice of the naïve T cells toward these distinct fates, cytokines produced by antigen presenting cells (APCs) exert powerful effects that promote or restrict these choices [4], [8], [9], [10], [11], [12]. Of the cytokines that regulate CD4^+^ T cell development, interferon-γ (IFN-γ) and IL-12 promote Th1 development, IL-4 promotes Th2 development, and in their absence, the cytokines IL-6 and TGF-β induce Th17 development. Cytokine-induced regulation of Th1, Th2 and Th17 development is mediated through the transcription factors T-bet [13], [14], [15], GATA-3 [16], [17], [18], [19], [20] and RORγt [21], respectively. In addition to cytokine signaling pathways, many other factors have been proposed to regulate these choices [6], [22], [23], [24], [25], [26], [27], [28].

Recent studies have suggested that distinct Notch ligands expressed on APCs might regulate Th1 and Th2 fate choice [29], [30], [31], [32]. Notch proteins are membrane-bound receptors that regulate diverse cell fate decisions in multi-cellular organisms [33]. Notch signaling regulates developmental processes during hematopoiesis and lymphopoiesis, and is essential for differentiation of single-positive T-cells from the common lymphoid progenitor [34], [35], [36], [37], [38], [39], [40]. Despite the assumption that manipulation of this linear pathway by different strategies should lead to a similar set of observations, the role of Notch signaling in Th1 and Th2 development has been controversial (Table S1A & B) [29]. Particularly provocative were reports that DLL1 biased naïve CD4^+^ T cells towards the Th1 fate [31], whereas Jag1 biased toward Th2 [30], suggesting that pathogens drive distinct T helper fate choices through the induction of alternative Notch ligands on antigen presenting cells [30].

While the induction of Th1 or Th2 development by distinct Notch ligands might resemble the mechanism of fate induction mediated by cytokines such as IL-12 and IL-4, it is difficult to explain how activation of Notch receptors by its ligands could instruct divergent fates. Understanding this difficulty requires an appreciation of how Notch is activated: binding of DLL1 or Jag1 ligands to the Notch extra-cellular domain triggers a conformational change that exposes a β-strand of Notch to cleavage by ADAM family metalloproteases [41], [42]. This cleavage results in shedding of the ectodomain [43], [44], generating an intermediate that is recognized by Nicastrin [45], a component of the enzyme γ-secretase. Nicastrin then transfers truncated Notch into the active site of γ-secretase, which cleaves the Notch transmembrane domain near the inner leaflet [46], [47]. Following this cleavage, the Notch intracellular domain (NICD) translocates to the nucleus where it regulates gene expression [47], [48]. The four mammalian Notch receptors regulate transcription through a common DNA binding protein, RBP-J [49], and require the recruitment of mastermind-like (MAML) proteins [50], [51], [52], [53] and additional co-activators [54], [55] to initiate transcription on target promoters. Given this activation mechanism, it is unclear how NICD could retain the memory of which ligand induced ectodomain shedding and translate this memory into distinct transcription profiles. Therefore, it is immensely interesting to explore the basis of such “ligand memory” and to explain how DLL1 could induce Th1 through T-bet upregulation [31], whereas Jag1 could initiate Th2 development by inducing GATA-3/IL-4 expression [30], [56], [57], when both ligands should lead to essentially the same intracellular signal within the T cells.

We examined the activity of Notch ligands in directing Th1/Th2 differentiation. In contrast to previous reports [30], [31], we demonstrate that DLL1 and Jag1 are insufficient for instructing specification of either Th1 or Th2 fates in the absence of polarizing cytokines, and can mildly enhance cytokine-induced Th1/Th2 responses. In addition, we show for the first time that Jag1 is incapable of activating Notch1 signaling in naïve CD4^+^ T cells, which express Lunatic Fringe. We also examined the requirement for Notch signaling on CD4^+^ T cell fate specification by removal of *Presenilin1 (PS-1)* and *PS-2* genes, which encode the γ-secretase catalytic subunits, and by removal of the nuclear Notch co-activator *RBP-J*, to separate Notch-independent from Notch dependent activities of γ-secretase.

First, we find that Notch signaling is not necessary for cytokine-induced Th1/Th2 fate selection, consistent with some previous studies that identified a co-stimulatory role for Notch [58], [59], [60]. This analysis, however, uncovered two novel, RBP-J-independent functions of presenilin, one contributing to the proliferative response and the other to secretion of cytokines in T-helper cells. Taken together, our data suggest that intact Notch signaling and Presenilins function permit optimal peripheral T-helper cell responses, rather than exerting direct influences on Th1/Th2 differentiation choices.

## Materials and Methods

### Mice

**Figure 1:**
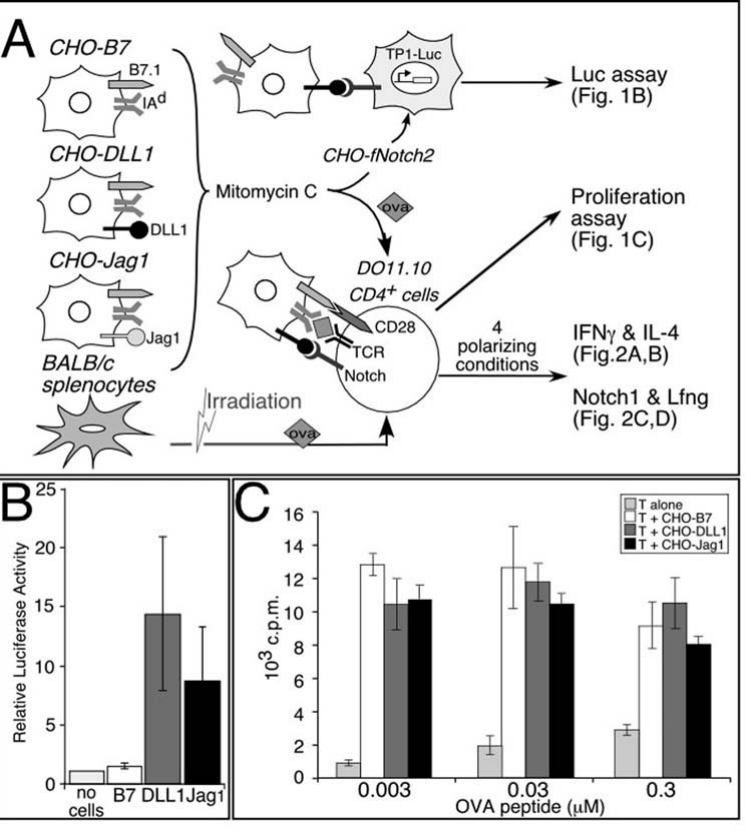
Functional Notch ligands on APCs do not affect APC-mediated T cell proliferation. (A) Schematic of the experiment. Artificial APC lines were treated with mitomycin C (100 µg/ml for 1 h) and seeded either for co-culture reporter assay or for activating naïve CD4^+^ T cells isolated from DO11.10 mice. Irradiated BALB/c spleen cells were used as the natural APC control. Activated CD4^+^ T cells were assayed for the rate of proliferation, level of IFNγ and IL-4 production under the 4 polarizing conditions, and the presence of activated Notch1 and Lfng proteins. (B) The Notch ligands expressed on APC lines elicit Notch2 cleavage and RBP-J-dependent transcriptional activation of *TP1*-luciferase in co-culture system. Results are mean±S.D. of three independent experiments. (C) Functional Notch ligands on APC lines do not affect T cell proliferation. Naïve CD4^+^ T cells purified from DO11.10 mice were cultured with the different APC lines under various concentrations of OVA peptide, as indicated on the horizontal axis. The cultures were pulsed with [^3^H] thymidine at 48 h, harvested and analyzed at 60 h. Data represent c.p.m±s.d. from triplicate wells.

**Figure 2 pone-0002823-g002:**
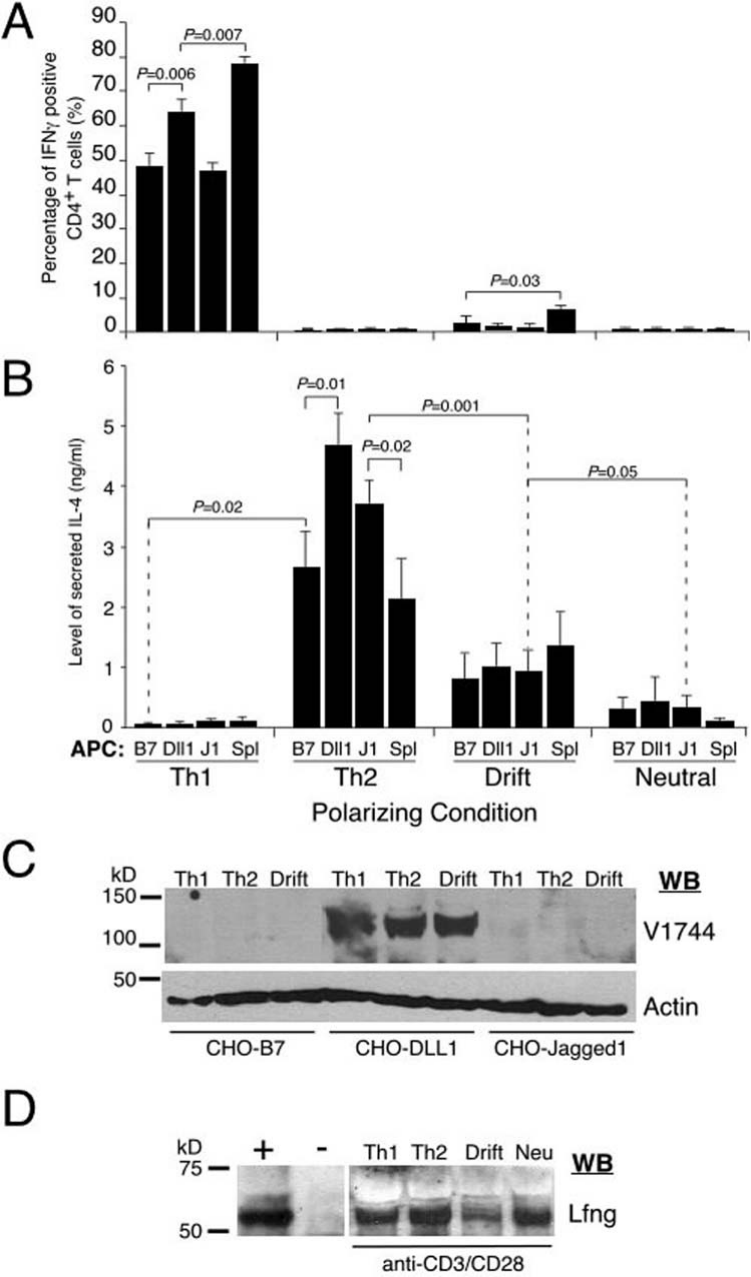
Notch ligands cannot instruct Th1/Th2 differentiation in the absence of inducing cytokines but only act selectively in co-stimulation. (A) Notch ligands cannot induce a Th1 program under “drift” or “neutral” conditions, whereas only DLL-1 enhances IFN-γ production under Th1 polarizing conditions. Naïve CD4^+^ T cells from DO11.10 mice were purified to >99% purity by a two-step protocol, and primed with different APC lines and 0.3 µM OVA peptide in 4 polarizing conditions for 2 days. Activated T cells were expanded in fresh media for another 5 days, re-stimulated with 4 h of PMA/Ionomycin and stained for intracellular IFN-γ. B7: CHO-B7, DLL1: CHO-DLL1, J1: CHO-Jag1 and Spl: BALB/c spleen cells. Results are mean±s.d. from three independent experiments. (B) DLL1 and Jag1 ligands do not cause significant differences in the level of IL-4 production under “drift” and “neutral” conditions. They only marginally enhance IL-4 cytokine secretion under Th2 polarizing conditions. Activated T cells were re-stimulated on Day 7 with anti-CD3 antibody for 24 h and before supernatant collection and ELISA. Results are mean±s.d. of three independent experiments. The *P* value was determined by student two-tailed *t* test. (C) Only CHO-DLL1 APC line triggers Notch1 cleavage in activated CD4^+^ T cells. Naïve CD4^+^ T cells from DO10.11 mice were purified to >99% purity by the two-step protocol and primed with various APC lines and 0.3 µM OVA peptide in 3 different polarizing conditions. They were isolated 24 h later and probed with V1774 and actin antibodies. WB: western blot. (D) Detection of Lunatic Fringe in CD4^+^ T cells. Naïve CD4^+^ T cells purified from DO11.10 mice were activated with anti-CD3/CD28 antibodies under the 4 polarizing conditions for 24 h and harvested for western with anti-Lfng antibody. Lysate from newborn pup (P1) was used as positive control whereas negative control was lysate from NIH3T3 cells. WB: western blot.

**Figure 3 pone-0002823-g003:**
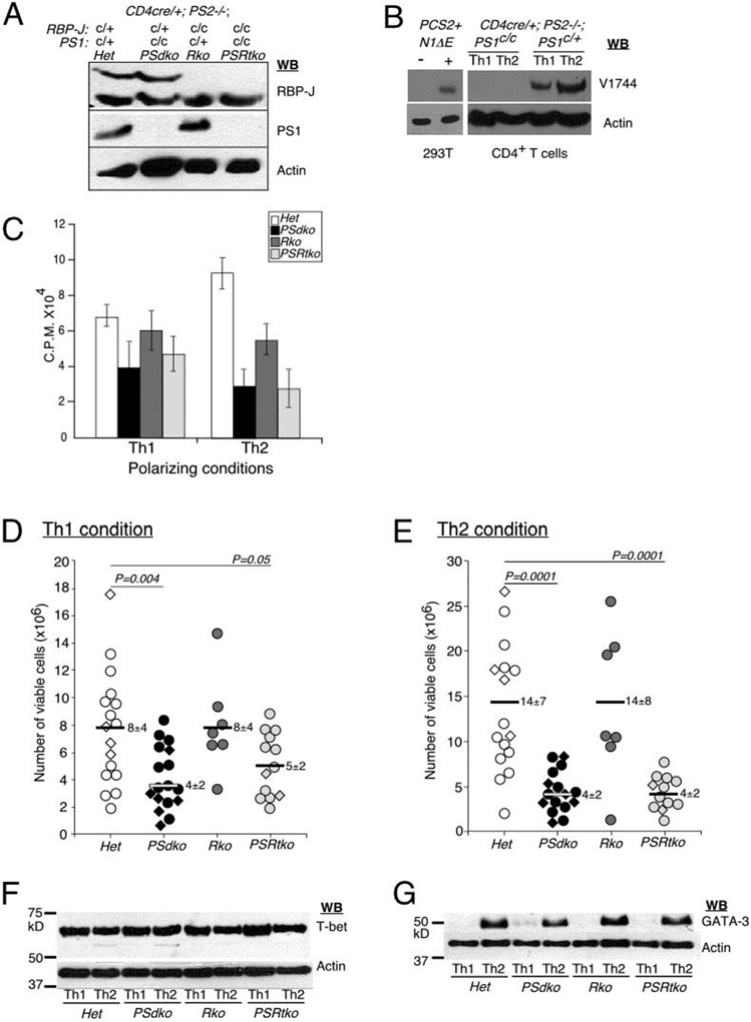
RBP-J independent function of presenilin is required for optimal T cell expansion. (A) Efficient deletion of *Presenilin1* and *RBP-J* alleles by *CD4-cre* transgene. Naïve CD4^+^ T cells isolated from conditional knockout littermates (n≥7 animals per genotype) showed no detectable level of RBP-J and presenilin1 proteins. WB: western blot. (B) Removal of PS1/PS2 proteins abolished Notch1 signaling in activated naïve CD4^+^ T cells. Activated Notch1 is detected in activated control CD4^+^ T cells under both Th1 and Th2 polarizing conditions but not detected in T cells that have targeted ablation of *PS1/PS2* alleles. Non transfected HEK293T cells were used as negative control, while cells transfected with PCS2+N1ΔE was used as a positive control. CD4^+^ T cells were isolated with anti-CD4 magnetic beads on MACS column to >95% purity from the spleens of 2 months old littermates. This protocol allows co-purification of natural APCs that provide the Notch ligands (compare with Figure 2C where no Notch1 activation was observed when a two-step purification method was used). T cells were activated with anti-CD3/CD28 in Th1 or Th2 polarizing conditions for 24 h. T cells were then FACS sorted for CD4^+^ population and probed with V1744 antibody. WB: western blot. (C) Proliferation capacity was measured by ^3^[H]-thymidine incorporation. Reduced proliferation was observed in *PSdko* and *PSRtko* cells under both Th1 and Th2 polarizing conditions. *Rko* cells were not significantly different than controls. Results are presented as mean±S.D. of five wells and representative of at least three independent experiments. (D, E) Reduction in the final number of viable *PSdko* and *PSRtko* T cells 6 days after activation. Naïve CD4^+^ T cells from different genotypes were stimulated with anti-CD3/CD28 antibodies for 2 days in Th1 and Th2 conditions before they were expanded in fresh media containing IL-2 cytokine. After 6 days of culture, T cells were harvested and viable cells were counted. Each circle/diamond indicates data of individual mouse. In Figures 1–4, circle (○) indicates data point in which CD4^+^ T cells were expanded by regimen 2 (Supplemental Table 4B), whereas diamond (◊) indicates result in which T cells were expanded according to regimen 3 (Supplemental Table 4C). The *P* value was determined by student two-tailed *t* test. (F, G) The expression of T-bet and GATA-3 is unaffected by the removal of RBP-J and/or presenilins. T cells activated in Th1 or Th2 conditions were harvested and probed with T-bet or GATA-3 antibodies after 6 days in culture. Results are representative of three independent experiments. WB: western blot.

All animal were housed and all experiments were conducted according to the IACUC guidelines and approved by the Washington University Animal Studies Committee. BALB/c (Charles River Labs), CD4-cre^Tg/Tg^ (C57BL/6; Taconic) [61], PS1^C/C^ PS2^−/−^ [62]; RBP-J^C/C^ [63] and DO11.10 TCR transgenic mice [64] used for the experiments in Figure 1–2, were described before. The parental PS1^C/C^ PS2^−/−^, maintained as C57BL/6/CD1 hybrids, was crossed with *CD4-cre^Tg/Tg^* and F1 offspring were backcrossed twice into *PS1^C/C^ PS2^−/−^* to obtain the genotypes *CD4-Cre^Tg/+^*, *PS1-1^C/+^*, *PS2^−/−^* (*Het*) and *CD4-Cre^Tg/+^*, *PS1-1^C/C^*, *PS2^−/−^ (PSdko)*. The *PS1/PS2* and *RBP-J* triple knockout mice were generated by first crossing *CD4-Cre^Tg/+^*, *PS1-1^C/+^*, *PS2^−/−^* mice with *PS1-1^C/C^*, *PS2^−/−^*, *RBP- J^C/C^* mice to generate *CD4-Cre^Tg/+^*, *PS1-1^C/+^*, *PS2^−/−^*, *RBP-J^C/+^* mice. These F1 offspring were crossed again with *PS1-1^C/C^*, *PS2^−/−^*, *RBP- J^C/C^* mice to produce F2 littermates with the following genotypes: *CD4-Cre^Tg/+^*, *PS1-1^C/+^*, *PS2^−/−^*, *RBP-J^C/+^(Het)*, *CD4-Cre^Tg/+^*, *PS1-1^C/C^*, *PS2^−/−^*, *RBP-J^C/+^* (*PSdko*), *CD4-Cre^Tg/+^*, *PS1-1^C/+^*, *PS2^−/−^*, *RBP-J^C/C^* (*Rko*) and *CD4-Cre^Tg/+^*, *PS1-1^C/C^*, *PS2^−/−^*, *RBP-J^C/C^* (*PSRtko*), which were used for the experiments in Figure 3–6.

**Figure 4 pone-0002823-g004:**
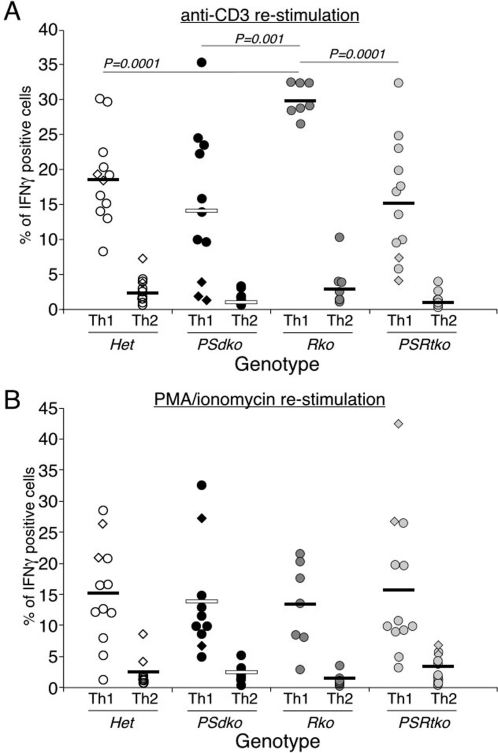
RBP-J and presenilins are not required for the production of intracellular IFN-γ in CD4^+^ T cells activated under Th1 polarizing conditions. (A, B) Production of intracellular INF-γ is unaffected in different mutant genotypes in Th1 polarizing conditions when compared to Th2 polarizing conditions. Elevated levels of IFN-γ production are detected by ICS in RBP-J-deficient cells under Th1 polarizing conditions after re-stimulation with anti-CD3 but not with PMA/Ionomycin. Naïve CD4^+^ T cells from different genotypes were stimulated with anti-CD3/CD28 antibodies for 2 days in Th1 and Th2 conditions. Cells were then expanded into new media containing IL-2 cytokine. T cells were harvested and counted after 6 days of culture. An equal number of T cells were re-stimulated overnight with (A) anti-CD3 antibody, or (B) PMA/Ionomycin. Brefeldin A was added in the final 4 h of stimulation before intracellular staining. Each circle/diamond represents data of individual mouse. The *P* value was determined by two-tailed *t* test.

**Figure 5 pone-0002823-g005:**
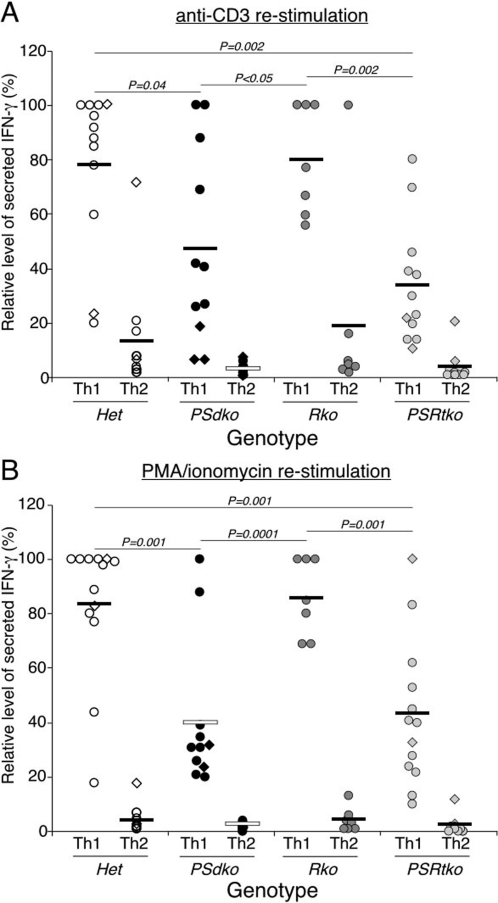
RBP-J-independent function of presenilin is required for optimal IFN-γ secretion from differentiated Th1 cells. (A, B) Impaired IFNγ cytokine production by PS1/PS2-deficient T cells is not rescued by the compound loss of RBP-J. The experiments were conducted as described in Figure 4 except that the supernatant was harvested for ELISA analyses. Each data point indicates individual mouse and is presented as the relative level of IFN-γ secretion. This value is determined by calculating the percent of IFN-γ secretion from individual mouse over the maximum level attained in each separate experiment (see Materials and Methods for details). The *P* value was determined by two-tailed paired *t* test.

**Figure 6:**
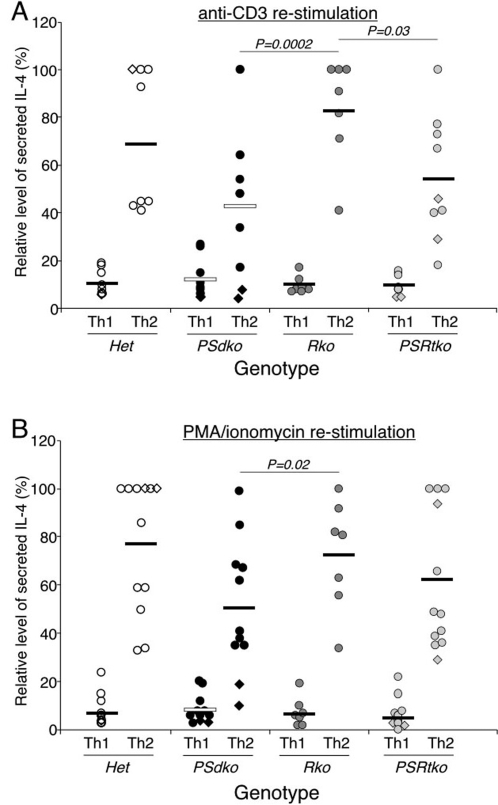
Reduced level of IL-4 secretion from PS1/PS2 and not RBP-J deficient Th2 cells. (A, B) PS1/PS2-deficient T cells have significant reduction in IL-4 cytokine production when compared to RBP-J-deficient T cells independent of the mode of re-stimulation. The experiments were conducted as described in Figures 5. Data is presented as the relative level of IL-4, determined by comparing the IL-4 secretion from individual mouse over the maximum level attained in each separate experiment. The *P* value was determined by two-tailed paired *t* test.

### Plasmids and retroviral constructs

PCS2+N1ΔE encodes truncated Notch1 protein that is a constitutive substrate of γ-secretase [44]. *TP1*-luc construct (PGa981-6) is a Notch reporter cassette that contains 12 tandem repeats of CSL binding sites upstream of luciferase [65]. pYITG and pCGP plasmids used for packaging retrovirus were a generous gift from Dr. W. Sha (University of California, Berkeley, CA). Mouse DLL1 cDNA clone (#9021250) was obtained from ATCC (Manassas, VA) and mouse Jag1 cDNA clone (#97002RG) was obtained from Invitrogen (Carlsbad, CA).

The primers 5′-CGGGATCCGCCAATGCGGTCCCCACGGACGCGC-3′ and 5′-GTTCTCGAGCTATACGATGTATTCCATCCGGTT-3 were used to amplify Jagged1 cDNA. The primers 5′-GAGGATCCGCCATGGGCCGTCGGAGCGCGCTAG-3′ and 5′-TTACTCGAGTTACACCTCAGTCGCTATAACAC-3′ were used to amplify DLL1 cDNA. The PCR amplified fragments were first blunt cloned into PCS2+ vectors and then replaced with original cDNA sequences by restriction digest. PCS2+DLL1 and PCS2+Jagged1 were then completely sequenced, digested with BamHI and XhoI, and the ligands subcloned into the Bgl II and XhoI site on the IRES-GFP-RV vector.

### Cell lines

CHO cell lines stably expressing either full-length Notch2 (CHOfNotch2) or full-length Delta-like-1 (fD1-CHO) were a generous gift from Drs. S. Chiba and H. Hirai (University of Tokyo, Japan). Parental CHO cells expressing MHC class II molecules (I-A^d^ haplotype) and B7-1 were a generous gift from Dr. A. Sharpe (Harvard University, Boston, MA) and were used to generate artificial APC lines that express Notch ligands. Retroviral infection of CHO cells was performed with a protocol modified from Dr. G. Nolan's lab (Stanford University, CA). Briefly, retrovirus was packaged by transfection of 293T cells with constructs pYITG, pCGP and the viral vectors using the calcium phosphate method (in BES buffered saline with chloroquine at a final concentration of 25 µM). After 9 h, transfected 293T cells were washed once and replenished with new media. Virion-containing supernatant (10 ml) was harvested 48 h post-transfection, filtered (0.45 µM) and transferred to CHO cells (4.5 ml of 293T supernatant in 6.5 ml of media supplemented with polybrene at a final concentration of 5 µg/ml). After 48 h, 6 to 25% of cells were infected. Three different GFP^hi^ APC lines (IRES-GFP-RV infected or CHO-B7; DLL1-IRES-GFP-RV infected or CHO-DLL1; Jagged1-IRES-GFP-RV infected or CHO-Jag1) were FACS sorted to >95% purity, stained with I-A^d^ and B7-1-phycoerythrin and analyzed by flow cytometry to confirm a comparable level of staining for MHC and B7 ligands (80–90% of the GFP^hi^ population; Figure S1A). All APC cells were maintained in Iscove's DMEM, supplemented with 10% heat-inactivated FBS, nonessential amino acids, sodium pyruvate, penicillin/streptomycin and β-mercaptoethanol. 293 T cells were maintained in DMEM media according to ATCC protocol.

### Co-culturing Experiment

1.2×10^5^ of CHOfNotch2 cells was seeded on 24-well plate 24 h prior to transfection. PCS2+βgal (control for transfection), *TP1*-luc and PCS2+ (as DNA carrier) plasmids were transfected by Lipofectamine™ 2000 according to manufacturer protocol. Artifical APC lines or fD1-CHO (positive control, at 0.1×10^6^) were added 24 hr after the transfection and luciferase assay was carried out 48 h later.

### Luciferase Assays

Cells were harvested after 48 h in co-culture and washed once with phosphate-buffered saline (PBS). Cells were lysed in 100 µl of lysis buffer (100 mM KPO_4_ buffer, pH 7.8; 0.2% Triton; 1 mM dithiothreitol (DTT); protease inhibitors) at room temperature for 10 min. 5 µl of lysate was used to determine β-galactosidase concentration (to normalize for transfection efficiency) according to the Tropix Galacton chemiluminescent substrate instructions. 50 µl of lysate incubated with luciferin assay buffer (30 mM Tricine, pH 7.8; 3 mM ATP; 15 mM MgSO_4_; 10 mM DTT; 0.2 mM CoA; 1 mM luciferin) was used to determine luciferase activity using a Tropix TR717 luminometer.

### Western Blot

Laemmli SDS sample buffer (+10 mM DTT) was added directly to half a million naïve CD4^+^ T cells and the mixture was heated at 55°C for 10 min. Protein samples were resolved by 12% (PS1), 8% (RBP-J, Lfng, GATA-3, T-bet, β-actin) and 6% (cleaved Notch1) SDS-PAGE gel in 25 mM Tris, 192 mM glycine, 0.1% SDS buffer. Proteins were then transferred to nitrocellulose in 25 mM Tris, 192 mM glycine, 20% methanol buffer. Blots were incubated with primary antibody in 0.1% Tween and 5% milk in PBS at 4°C overnight. The primary antibodies used were anti-β-actin at 1∶5000 dilution (Sigma, A5441); anti-V1744 at 1∶500 (Cell signaling, #2421); anti-RBP-J at 1∶100 (Cosmobio, SIM-2ZRBP2); anti-PS1 at 1∶1000 (sc-7860); anti-Lfng (sc-8239) at 1∶1000; anti-T-bet (sc-21749) at 1∶200; and anti-GATA-3 (sc-268) at 1∶200 (all from Santa Cruz). After three washes, the membranes were incubated with secondary antibody diluted 1∶5000 in 0.1% Tween PBS for 1 h at room temperature. The secondary antibodies used were anti-mouse (Amersham, NA931), anti-rabbit (Amersham, NA934) or anti-goat (Santa Cruz, sc-2020) IgG, horseradish peroxidase-linked species-specific antibodies. After three rinses, the protein was visualized with Super-Signal ® West Pico/Dura/Femto Chemiluminescent kit (Pierce) as per the Hyperfilm™ MP instructions (Amersham Biosciences).

### T cell purification and in vitro differentiation Naïve CD4^+^ T cells Purification

Two different methods were used in this study. The first method, used to generate Figure 3B, employed MACS column and anti-CD4 magnetic beads (Miltenyi Biotech, Auburn, CA) for rapid separation of CD4^+^ T cells. For other experiments in this study, a two-step purification protocol was used. The CD19+ fraction was first removed with anti-CD19 magnetic beads on MACS column (Miltenyi Biotech, Auburn, CA). Subsequently, the CD19-negative fraction was FACS sorted (MoFlo™, Dako) using CD4-FITC (Caltag, RM2501) and CD62L-phycoerythrin antibodies (Caltag, RM4304) to purify CD4^hi^, CD62L^hi^ population.

### Priming by APC

2×10^6^ artificial APC cell lines were seeded on a 60-mm culture plate one day prior to the experiment. On the day of the experiment, APC cells were treated with 100 µg/ml of mitomycin C (Sigma) at 37°C for 1 h, washed twice in PBS and lifted with 0.2 M EDTA. APCs were then seeded (at 0.25×10^6^) on 48-well plates (for subsequent activation of T cells) or added (at 0.1×10^6^) to transfected CHOfNotch2 (for reporter assay). To control for the activity of natural APCs, 5×10^6^ irradiated (2000 Rad) splenocytes from BALB/c mice (spl) were used. 0.5×10^6^ naïve CD4+ T cells isolated to >98% purity from DO 11.10 mice were added to the 0.25×10^6^ APCs in the presence of 0.3 µM OVA peptide and under one of the 4 polarizing conditions. **Th1:** 10 U/ml of IL-12 and 10 µg/ml of anti-IL-4 (11B11). **Th2:** 100 U/ml of IL-4, 3 µg/ml of anti-IL-12 (TOSH) and 10 µg/ml of anti-IFNγ (H22). **Drift:** Only media. **Neutral:** 10 µg/ml of anti-IL-4 (11B11), 3 µg/ml of anti-IL-12 (TOSH) and 10 µg/ml of anti-IFNγ (H22). Polarization experiments were carried out in a 7-day cycle, starting with activation on day 0. On day 2, cells were expanded into fresh media containing 40 U/ml of IL-2 (see Table S4A for additional details). T cells were collected on day 7 and counted. 0.5×10^6^ T cells were then re-stimulated either with plate-bound anti-CD3 for 24 h and its supernatant collected for ELISA, or PMA (50 ng/ml) and Ionomycin (1 µM) in the presence of Brefeldin A (1 µg/ml, Epicenter Technology, Epicenter Technologies, Madison, WI) for the 4 h and subjected to intracellular cytokine staining.

### Priming by anti-CD3/anti-CD28

48 well plates were coated with anti-CD3 (500A2: 1 µg/ml) and anti-CD28 (0.8 µg/ml) overnight at 4°C. 0.5×10^6^ of purified naïve CD4^+^ T cells (>95%) from different genotypes (*Het*, *PSdko*, *Rko & PSRtko*) were stimulated in 6-day cycles, starting with activation on day 0 in either Th1 or Th2 polarizing conditions (see below). On day 2, cells were expanded into fresh media plus 40 U/ml of IL-2 (see Table S4B and C for details) and resting cells were collected on day 6/7 and counted. 0.5×10^6^ of cells from each genotype were re-stimulated overnight with plate-bound anti-CD3 or PMA (50 ng/ml) and Ionomycin (1 µM). Brefeldin A (1 µg/ml; Epicentre Technology) was added for the final 4 h of each stimulation. Cells were subjected to intracellular cytokine staining and supernatant collected for ELISA.

### Proliferation Assay

0.1×10^6^ purified T cells were plated onto a 96-well plate seeded with an equal number of APC lines (Figure 1C & Figure S1C) or pre-coated with anti-CD3/CD28 antibodies (Figure 3C) in 100 µl media. After 48 h, cells were pulsed for 12 h with 1 µCi/well of [^3^H]-thymidine.

### Intracellular cytokine staining, ELISA and FACS analysis

After re-stimulation, the cells harvested were resuspended in FACS buffer (3% FCS in PBS, 0.01% azide). Cells were stained with phycoerythrin (PE)-cy7 conjugated anti-CD4 antibody (552775, BD Bioscience Pharmingen™, San Diego, CA) and fixed in 2% formaldehyde for 15 min at room temperature. After washing once with PBS, cells were permeabilized twice with saponin (0.05% followed by 0.5% in FACS buffer). The cells were stained with respective antibodies in 0.5% saponin at 4°C for 30 min. Isotype control and cytokine antibodies used were from BD Bioscience Pharmingen™ (San Diego, CA). The PE-conjugated antibodies are: anti-IL4 (554389), anti-IFNγ (554412), Rat IgG1κ (554685) and Rat IgG2bκ (556925). The APC-conjugated antibodies are: anti-IL4 (554436), anti-IFNγ (554413) and Rat IgG1κ (554686). Cells were then washed once and resuspended in FACS buffer for analyses. ELISA (enzyme-linked immunosorbent assay) was carried out with mouse Th1/Th2 cytokine cytometric bead array according to manufacturer's protocol (BD Bioscience, San Diego, CA) and analyzed on a FACSCaliber (Becton Dickinson, San Jose, CA).

### Tabulation of cytokine production

Ten independent *in vitro* polarization experiments were conducted for Figures 4–6, comprising of at least one control (*Het*) and two test genotypes (*PSdko*, *Rko or PSRtko*) per experiment. The level of secreted cytokines was measured empirically. In each experiment, the highest secreted cytokine level was set as 100% and used to normalize the values from other genotypes. Results from the independent experiments were compiled with these percentiles and presented as relative cytokine level. The normality of distribution and significance were calculated by Kolmogorov-Smirnov and two-tailed paired *t*-test respectively.

## Results

### Notch ligands expressed by APCs are functional and do not alter T cell proliferation

Based on *in vitro* differentiation assays of naïve CD4^+^ T cells, an inductive role for Notch in promoting Th1 or Th2 development was recently suggested [30]. To test how Notch signals could induce different T-helper cell differentiation programs, we created Notch-ligand expressing APCs by modifying Chinese hamster ovary (CHO) cells that stably express B7-1 and MHC class II (I-A^d^) to also express either DLL1 or Jag1 ligands (Figure 1A and Figure S1A). To confirm the effectiveness of Notch ligands expressed by these APCs, we co-cultured our panel of APC lines with CHOfNotch2 reporter cells that express a full-length Notch2 receptor [66] and the *TP-1* luciferase reporter cassette [65]. We found that the APCs that express DLL1 or Jag1 could robustly stimulate luciferase activity in CHOfNotch2 cells. In contrast, the APC line CHO-B7, which contains GFP-expressing retroviral vector and lacks Notch ligands, failed to induce luciferase activity in CHOfNotch2 cells (Figure 1B). These results are an important positive control demonstrating the functional integrity of the DLL1 and Jag1 ligands expressed by our APCs.

Given that attaining optimal T-helper differentiation requires proliferation [67], [68], [69], [70], we tested the proliferative responses of naïve CD4^+^ T cells activated by APCs with or without Notch ligands. To inhibit APC proliferation, APC were exposed to 1 hr of 100 µg/ml mitomycin C treatment. This regimen effectively blocked APC proliferation; allowed Notch ligands on the APC lines to induce luciferase activity in CHOfNotch2reporter lines (Figure 1B); and triggered proliferative responses in naïve DO11.10 CD4^+^ T exposed to three different concentrations of OVA peptide (Figure 1C). These results indicated that activation of Notch by APCs in the presence of CD28 co-stimulation did not enhance antigen-driven T cell proliferation. More importantly, this system provides us with the ability to test whether functional Notch ligands on APCs can instruct Th1 or Th2 fate selection.

### Functional Notch ligands expressed by APCs do not instruct T cell differentiation

To examine the role of Notch ligands on T cell differentiation, naïve DO11.10 CD4^+^ T cells were activated with these three APC cell lines under 4 conditions (Figure 1A). We included both Th1- and Th2-polarizing conditions to test if either ligand could augment or inhibit cytokine-driven differentiation. We also included two kinds of non-polarizing conditions to test if Notch ligands themselves were sufficient to bias/instruct differentiation: in one, cytokines are neither added nor neutralized to test whether Notch ligands can bias toward Th1 or Th2 differentiation. If they could induce IL4, for example, this condition will allow for auto-stimulation. In the second type of non-polarizing condition, polarizing cytokines are neutralized to test whether Notch ligands can induce Th1 or Th2 fate acquisition on their own. Finally, we used irradiated BALB/c splenocytes as APCs under all conditions as a positive control for normal cytokine-induced Th1 and Th2 differentiation.

Activated T cells were passage 7 days (detailed in Table S4A), harvested and counted. Equal number of viable cells was re-stimulated with PMA/ionomycin (4 h in the presence of BFA for intracellular staining) or anti-CD3 (for ELISA of the supernatant). Artificial APC lines induced T cells expansion 2 to 3.6 fold greater than irradiated splenocytes under all conditions (Figure S1C). These results indicated that our APC lines are comparable to (if not better than) natural APCs in priming naïve CD4^+^ T cells.

Under Th1 polarizing conditions, we found high levels of intracellular IFN-γ production in CD4^+^ T cells stimulated with any APC line, regardless of Notch ligand expression (Figure 2A; individual data points were depicted in Figure S2A-C & Table S2A). CD4^+^ T cells primed with splenocytes as APCs produced the highest percentage of IFNγ-positive cells detected by intracellular staining (ICS) (78%), closely followed by T cells primed with CHO-DLL1 (64%; P = 0.006) and by CHO-B7 or CHO-Jag1 (47–48%). Similar results were observed when secreted IFN-γ was measured by ELISA of the supernatant (detailed in Table S2B). Likewise, under Th2 polarizing conditions, high levels of IL-4 were produced by CD4^+^ T cells stimulated with any APC line, regardless of Notch ligand expression (Figure 2B, detailed in Table S3B). CD4^+^ T cells primed with CHO-DLL1 and CHO-Jag1 secreted slightly higher amounts of IL-4 compared to the CHO-B7 APC line or splenocytes. These results indicate that Notch ligands can augment cytokine-induced Th1 or Th2 differentiation but cannot interfere with this specification process. In particular, DLL1, suggested to induce Th1 development [30], [31], did not re-direct T cells toward a Th1 fate under Th2-polarizing conditions. Likewise, Jag1, suggested to induce Th2 development [30], did not re-direct T cells toward a Th2 fate under Th1-polarizing conditions.

Next we examined non-polarizing conditions of activation. Surprisingly, neither DLL1 nor Jag1 had a significant effect on Th1 or Th2 development under either of the non-polarizing conditions compared to the CHO-B7 APC line (Figure 2A, B, and Table S2, 3). When cytokines were neither added nor neutralized, DLL1 expression by APCs did not cause a significant increase in IFN-γ production, as would have been predicted from a previous study [30], [31]. Similarly, Jag1 expression on APCs did not increase IL-4 production, apparently excluding a role for Notch ligands in biasing Th1/Th2 fate choice. Finally, under conditions where polarizing cytokines were neutralized, neither DLL1 nor Jag1 led to significant changes in IFN-γ or IL-4 production (Figure 2A, B), indicating that these ligands are not sufficient for driving Th1 or Th2 fate choice. These data are inconsistent with the results reported for DLL1 and Jag1 expression on DCEK hi7 fibroblasts as artificial APCs [30], which claimed DLL1 and Jag1 to induce Th1 and Th2 development, respectively. Thus, our results would appear to exclude an instructive role for Notch ligands in inducing actual Th1/Th2 development from naïve CD4^+^ T cells.

The inability of Notch ligands expressed by our APC lines to induce Th1 or Th2 differentiation could have resulted from their inability to activate Notch signaling in naïve CD4^+^ T cells, despite their demonstrated activity in reporter cells (Figure 1B). Thus, we tested if Notch1 activation occurred in naïve CD4^+^ T cells co-cultured with CHO-B7 APC, CHO-DLL1 or CHO-Jag1 by directly measuring the production of NICD1. We found that robust Notch1 activation was induced in CD4^+^ T cells by CHO-DLL1, but not by the CHO-B7 cell line, as expected (Figure 2C). However, we found no evidence of Notch1 activation in CD4^+^ T cells induced by CHO-Jag1 cells (Figure 2C). Lunatic Fringe (Lfng) can modify the glycosylation pattern of Notch1 receptors (but not Notch2) in a manner that potentiates DLL1-mediated signaling but inhibits Jag1-mediated signaling [71], [72], [73]. The inability of Jag1 to activate Notch1 in CD4^+^ T cells but to activate Notch2 signaling in a reporter line (Figure 1B) would be consistent with Lfng activity rendering their Notch1 receptor insensitive to activation by Jag1. To test this, we measured Lfng protein expression by Western analysis in activated naïve CD4^+^ T cells (Figure 2D). Notably, Lfng protein was easily detected in CD4^+^ T cells under all assay conditions as well as naïve CD4^+^ T cells from different genetic backgrounds (data not shown). This result could explain the observed lack of effect of Jag1 on Th1 differentiation. In summary, these results confirms that Notch signaling is activated by DLL1 in CD4^+^ T cells, yet this is insufficient to instruct either Th1 or Th2 fate specification, nor can NICD act to redirect CD4^+^ T cell differentiation under these conditions (Figure 2).

### Conditional removal of presenilin and RBP-J in CD4^+^ T cells

The results above demonstrated that Notch activation was not sufficient to instruct T-helper cell fate selection. To test for a genetic requirement for Notch signaling in CD4^+^ T differentiation along Th1/Th2 lineages, we used a conditional deletion approach to eliminate all presenilin (PS) activity in CD4^+^ T cells. This is similar to the approach taken in a recent study [74], except that different targeted *PS-1* and *PS-2* alleles [62] were used. Due to strain or allele differences that may have restricted CD4-Cre expression to the DP stage, we were able to obtain normal numbers of CD4^+^ T cells from all genotypes, in contrast to the observations reported by Laky and Fowlkes [74]. For our analysis, we used naïve CD4^+^ T cells isolated from mice of the genotypes *CD4-Cre^Tg/+^*, *PS1-1^C/+^*, *PS2^−/−^*, *RBP-J^c/+^(Het)*; *CD4-Cre^Tg/+^*, *PS1-1^C/C^*, *PS2^−/−^*, *RBP-J^c/+^ (PSdko)*; *CD4-Cre^Tg/+^*, *PS1-1^C/+^*, *PS2^−/−^*, *RBP-J^c/c^* (*Rko*) or *CD4-Cre^tg/+^*, and *PS1-1^C/C^*, *PS2^−/−^*, *RBP-J^c/c^* (*PSRtko*) (See Materials and Methods). We confirmed that complete deletion of targeted alleles occurred in naïve CD4^+^ T cells purified from *PSdko*, *Rko or PSRtko*, lacking expression of either PS-1 or RBP-J as expected (Figure 3A), whereas normal levels of PS-1 or RBP-J were expressed in heterozygote littermate controls. Next, we asked if Notch signaling is activated during the process of T cell activation in our system. In the presence of natural APCs, control CD4^+^ T cells showed evidence of Notch1 activation 24 h after stimulation, whereas *PSdko* CD4 T cells showed no accumulation of NICD1, indicating an absence of Notch activation (Figure 3B). These controls confirmed that Notch signaling is active during normal T cell activation and that our genetic manipulations have successfully eliminated Notch signaling as intended prior to activation of CD4^+^ T cell.

### An RBP-J independent activity of presenilin contributes to CD4^+^ T cell expansion

We next examined the proliferative capacity of activated CD4^+^ T cells under both Th1 and Th2 conditions at 48 h by H^3^-thymidine incorporation assay (Figure 3C). Over several independent experiments, Notch ligands did not enhance proliferation in the presence of B7 co-stimulation (Figure 1C), whereas presenilin-deficient (*PSdko*) and presenlin- and RBP-J-deficient (*PSRtko*) T cells consistently exhibited lower proliferation (Figure 3C). Consistent with the proliferation results, cellular expansion, measured by counting the number of viable cells 6 days after activation, was also significantly impaired in *PSdko* and *PSRtko* populations (Figure 3D & E). Specifically, the heterozygote Th1 cultures showed a mean cell number of 8×10^6^ cells, which was reduced to 4×10^6^ cells in *PSdko* cells. The heterozygote Th2 cultures, which showed a mean cell number of 14×10^6^ cells, was also reduced to 4×10^6^ cells in *PSdko* cells. A similar reduction in cellular expansion was observed when stimulated T cells were treated with γ-secretase inhibitors [58], [59]. In contrast, the deletion of *RBP-J* did not significantly alter cellular expansion in T cells (Figure 3D, E), in agreement with earlier observations [75]. Interestingly, we saw a significant reduction in cellular expansion of triple mutant (*PSRtko*) T cells lacking PS-1, PS-2 and RBP-J when compared to control T cells from heterozygous littermates (Figure 3D and E). This result revealed a requirement for presenilin, but not RBP-J, in T cell expansion, consistent with a role for either a Notch-independent function of γ-secretase, or an RBP-J-independent function of Notch. Given that Notch ligand did not alter the level of proliferation induced by OVA peptide (Figure 1C), a Notch-independent function of γ-secretase seems most likely to be required for optimal T cell proliferation.

We also examined the expression of the transcription factors T-bet and GATA-3 in these cells 6 days after activation (Figure 3F and G). Notably, we found that T-bet was expressed by all cells, regardless of genotype (Figure 3F). In contrast, GATA-3 was present only in T cells exposed to Th2 polarizing conditions, regardless of their genotype (Figure 3G). The persistence of T-bet and GATA-3 expression under Th1 or Th2 conditions indicated that cytokine stimulation was not dependent on intact Notch signaling. Since other methods of manipulating Notch signaling have been reported to influence these processes *in vitro* [30], [31], [56], [57], [76], [77], we next turned our attention to determining the effects of presenilin and RBP-J deficiency on Th1/Th2 fate selection under polarizing conditions.

### An RBP-J independent presenilin activity regulates the levels of Th1 and Th2 cytokine secretion

If the Notch pathway is linear, then deletion of any one of its components should cause the same effect as deletion of any other component. In particular, a linear Notch pathway would predict that deletion of RBP-J [30], [75] would result in similar changes as would Notch blockade by γ-secretase inhibitors, or even by over-expression of dominant-negative (DN) MAML [78]. However, evidence has been accumulated that this may not be the case. In particular, removal of RBP-J and over-expression of DN-MAML both inhibited Th2 differentiation, but γ-secretase inhibitors did not block Th2 differentiation, and instead inhibited Th1 differentiation [77]. Because RBP-J is associated with co-repressors in the absence of a Notch signal [79], this discrepancy was suggested to reflect de-repression of a critical target (e.g. T-bet; [77]). Likewise, activation of the Notch pathway has not led to the same result. For example, over-expression of NICD1 in CD4^+^ T cells induced T-bet in some studies but not in others [31], [77]. In contrast, NICD1 induced IL-4 and GATA-3 in other studies [30], [56], [57], [76]. Thus, there is evidence that the effects of Notch inhibition or activation may be context dependent, perhaps due to the existence of a bifurcation in the pathway.

For these reasons, we compared Th1 and Th2 development in CD4^+^ T cells that lacked presenilin or RBP-J proteins (Figure 4, 5, 6). We examined the ability of these T cells to generate IFN-γ by ICS following differentiation *in vitro* after activation under both conditions in response to either overnight anti-CD3 treatment (Figure 4A) or PMA/Ionomycin treatment (Figure 4B). The percent of IFN-γ expressing cells from individual mice is shown for each experiment as a circle or diamond, and the mean of all mice in one group is presented as a bar. First, IFN-γ is produced by T cells only under Th1 conditions, and not under Th2 conditions, for littermates from all genotypes. Second, the absence of presenilin or RBP-J did not compromise the acquisition of Th1 fate (assessed as the ability to express IFN-γ) under Th1 conditions, measured following re-stimulation with anti-CD3 or with PMA/Ionomycin. In agreement with previous reports, we see an increase in the percent of *RBP-J*-deficient T cells producing IFN-γ under Th1 conditions [30], [75]. Thus, RBP-J-deficient CD4^+^ T cells are capable of Th1 differentiation.

Although we observed a reduction in the percentage of IFN-γ positive, presenilin deficient (*PSdko*) T cells, this difference was not statistically significant when compared to CD4^+^ T cells isolated from heterozygous littermates. Thus, commitment to Th1 development was still functional in CD4^+^ T cells lacking presenilin activity (Figure 4), inconsistent with the observations based on pharmacologic inhibition of γ-secretase [77]. Furthermore, *PSdko* CD4^+^ T cells differentiated under Th1 conditions committed to IFN-γ production at a much higher frequency than when differentiated under Th2 conditions, demonstrating their ability to functionally respond to Th1-inducing stimuli. No significant difference was detected between *PSRtko* and heterozygous T cells, or between *PSdko* and *PSRtko* T cells (Figure 4A, B). In summary, as measured by ICS, cytokine-induced Th1 fate commitment does not require the canonical Notch pathway [78].

Next we examined the ability of these T cells to secrete IFN-γ under Th1 and Th2 conditions after re-stimulating an equal number of T cells by either anti-CD3 treatment (Figure 5A) or PMA/Ionomycin treatment (Figure 5B). The amount of IFN-γ secretion is shown for individual mice and the mean is presented as a horizontal bar. Generally, the results were similar to those obtained by intracellular cytokine staining, although quantitative differences are now evident. First, for all genotypes tested, IFN-γ secretion was always significantly higher in Th1 conditions compared to Th2 conditions, indicating that the commitment to Th1 development was generally intact in *PSdko* T cells and RBP-J-deficient (*Rko*) T cells. However, the amount of IFN-γ that was secreted from *PSdko* T cells was reduced by about half, compared to control T cells from heterozygous littermates (Figure 5A). In contrast, despite an increase in the number of IFN-γ positive cells (Figure 4A), IFN-γ secretion from RBP-J-deficient T cells was not statistically different from control T cells (Figure 5A). To explain the increase in Th1 commitment seen in RBP-J null cells, it was proposed that de-repression of the *T-bet* gene occurred when RBP-J was removed [77]. If true, removing RBP-J in presenilin-deficient T cells (*PSRtko*) would de-repress *T-bet* and thus restore IFN-γ secretion. Instead, *PSRtko* T cells also exhibited a similar reduction in IFN-γ secretion to *PSdko* cells when compared to control T cells, even though the level was still far higher than when they were activated under Th2 conditions. In summary, the overall level of Th1 commitment, as assessed by ICS, was not significantly reduced in *PSdko* or *PSRtko* cells; however, we observed a variable reduction (50–70%) in the magnitude of IFN-γ secretion from T cells deficient in presenilin compared to all other T cells. These results suggest that an RBP-J independent action of γ-secretase is necessary to achieve maximal secretion of IFNγ from Th1 cells, independent of whether anti-CD3 or PMA/Ionomycin were used for re-stimulation (Figure 5).

We also analyzed IL-4 secretion for these genotypes activated under both Th1 and Th2 conditions (Figure 6). IL-4 was produced by all genotypes of T cells selectively under Th2 conditions, indicating that Th2 fate specification was intact in both presenilin-deficient and RBP-J-deficient T cells. Further, RBP-J-deficient T cells showed no significant differences in IL-4 production compared to controls T cells from heterozygous littermates (Figure 6). Similar to what we saw with IFN-γ secretion by Th1 cells, IL-4 secretion from *PSdko* T cells were somewhat reduced compared to controls. Although this reduction was not statistically significant in comparison to heterozygous littermates, it was statistically significant in comparison with RBP-J deficient T cells (Figure 6A, B). Similar reduction (∼20–40%) was also seen in triple mutant *PSRtko* T cells. Thus, we uncovered a general, RBP-J independent action of presenilin or γ-secretase that contributes to the magnitude of cytokine secretion by differentiated Th1 and Th2 cells.

## Discussion

Examples of Notch-dependent decisions in the hematopoietic system include the development of Marginal Zone B cells [80], [81], [82], multiple steps during T-cell development [63], [83], [84], [85], [86], [87], [88], [89], [90], [91], and vascular development (reviewed in [92]). In the examples above, the dependence of the developmental decision on Notch is similarly revealed independently of the experimental design or genetic background. Due to the linearity of the Notch signaling pathway involved in these decisions, the outcome of Notch inhibition is not influenced by the step at which Notch inhibition occurs (e.g., ligand binding, γ-secretase cleavage, association with RBP-J, or assembly of the activation complex). In contrast, the contribution of Notch signaling to peripheral T cell differentiation remains highly controversial because different experimental approaches have resulted in strikingly different outcomes (Table S1)[29]. In particular, the finding most difficult to reconcile with known mechanisms of Notch activation was that specific Notch ligands were able to instruct distinct T-helper fates [30]. These results were provocative because they implied a mechanism of “ligand memory” by which different ligands could produce distinct NICD activities in the nucleus.

This study tested the proposal of “ligand memory” by re-creating artificial APCs that express functional Notch ligands DLL1 and Jag1 that were previously suggested to instruct Th1 or Th2 differentiation, respectively [30]. In our system, we provide clear evidence verifying the ability of Notch ligands to activate Notch-dependent transcription from one or more receptors. Moreover, our controlled experiments were capable of detecting a bias towards Th1 and Th2 differentiation when natural APCs were used (Figure 2A, B). Despite that, neither DLL1 nor Jag1 could instruct or redirect T-helper fate specification in this assay. Specifically, Notch1 and Notch2 activation occurred in response to DLL1, yet DLL1 could not induce Th1 nor inhibit Th2 differentiation (Figure 2C). Surprisingly, we discovered that Jag1 was incapable of activating Notch1 in naïve CD4^+^ T cells, most likely due to the presence of Lfng in naïve CD4^+^ T cells from several genetic backgrounds (Figure 2D and unpublished data). This finding further diminishes the likelihood that Jag1 plays a significant role in Th2 differentiation through Notch1 activation [71], [93]. Although Jag1 could still activate Notch2 in the presence of Lfng, previous studies indicated that NICD1, and not NICD2, triggered robust Th2 responses (Amsen et al., 2003, Amsen et al., 2007; Fang et al., 2007).

Some previous studies have induced Notch activation with immobilized Notch ligands [31]. One caveat is that we cannot directly compare the “level” of Notch activation produced by APC-expressed ligands with those produced by immobilized DLL1-Fc molecules [31]. Conceivably, higher levels of NICD were achieved in the study by Maekawa [31], which titrated the level of immobilized (i.e., non-physiological) ligand presented to the CD4^+^ T cells. Regarding the discrepancy between APCs used in different studies, it will be necessary to perform side-by-side comparisons to resolve this apparent paradox.

Instead of identifying a mechanism of “ligand memory” capable of instructing T-helper fate, we conclude that Notch is not capable of inducing the initial steps towards fate acquisition. Rather, our data suggest that Notch can, at most, cooperate with cytokines to optimize T-helper differentiation (Figure 2C & D), consistent with a co-stimulatory role that has been suggested previously [59], [60], [78]. The distinct transcriptional activities of NICD during Th1 or Th2 differentiation *in vitro* could be explained if accessibility to target gene loci was influenced by a previous exposure to cytokine-induced remodeling, independent of Notch ligands.

We find that removal of intact Notch signaling by targeted deletion of *Presenilins* and/or *RBP-J* genes does not prevent cytokine-induced Th1/Th2 fate specification (Figure 4, 5, 6). This finding would be inconsistent with a genetic requirement for Notch signaling during the initial fate selection. Since the T-helper differentiation program is poised for rapid execution in response to multiple cues, the choice of experimental system and the “strength” of the stimulus will impact the amplitude of the contribution Notch makes. Our results suggest that intact Notch signaling may act to allow cells to attain the maximum level of commitment when insufficiently stimulated, highlighted by the physiological requirement of RBP-J for optimal Th2 response in T cells primed by parasite–exposed APC [56]. Although these reports indicate that Notch signaling may augment GATA-3 transcription *in vivo*, they did not demonstrate a specific role for Jagged in this process [56], [57], nor did they ask if stronger T-cell activation would bypass the need for Notch, as would be predicted by a co-stimulatory function of Notch in this process.

The present study is one of a few studies that have compared the effects of disrupting the Notch pathway during T-helper differentiation at two independent positions in the pathway. Importantly however, this is the only study that has performed epistatic analysis of γ-secretase and RBP-J deficiencies in this system. As defective T cell expansion and cytokine production were not observed in the absence of RBP-J alone, this epistatic analysis has revealed unexpected RBP-J-independent (and perhaps Notch-independent) functions of presenilins in regulating these processes. One function, regulating optimal T cell expansion, was reported by others [58], [59]. Given that proliferation is necessary to attain optimal T-helper differentiation [67], [68], [69], [70], a defect in proliferation may have led to the misinterpretation of γ-secretase function in T-helper cell specification. The second function, observed under all the experimental conditions we deployed in this study, contributes to the production or secretion of cytokines from committed, differentiated T-helper cells. Since we have inactivated γ-secretase by deleting the *Presenilin genes*, one or both of the above functions may reflect a protease-independent activity of presenilin [94], [95]. Distinguishing between these two presenilin activities will require epistatic analyses with the other γ-secretase components, but that is beyond the scope of this study.

In summary, the data presented here are inconsistent with the instructive role of Notch in Th1/Th2 fate specification. Instead, separable functions of Notch and presenilin as genetic modifiers of T-helper cell differentiation pathway(s) can account for the dependence of published conclusions on genetic background and experimental systems [29]. Despite clues pointing to TCR activation as the pathway modified by Notch and/or presenilin [74], this remains an important open question that will have to be addressed experimentally.

## Supporting Information

Figure S1 Characterization of the artificial APCs lines that express Notch ligands (A) Expression of I-Ad and B7.1 on CHO cells. CHOfNotch2 is a CHO line that stably expresses full-length Notch2 receptor (Shimizu et al., 2000). CHO-I-Ad expresses I-Ad MHC molecule but not B7.1 ligand. The APC parental line used for the priming experiment contained both I-Ad & B7.1 molecules. CHO-B7 (control line) was generated by infection with empty GFP vector, CHO-DLL1 by infection with DLL1-ires-GFP-RV construct, and CHO-Jag1 with Jagged1-ires-GFP-RV construct. Left: Staining with PE-conjugated I-Ad antibody. Right: Staining with PE-conjugated B7-1 antibody. X-axis is GFP signal. (B) Luciferase assay with co-culture experiments indicates comparable Notch ligand activity in our APCs lines. CHOfNotch2 cells was transfected with TP1-luciferase and PCS2+βgal constructs for 24 h before co-culturing with different ligands expressing cells. The fD1-CHO cell, a published line that exhibits functional ligand activity (Shimizu et al., 2000), was used a positive control in the experiment. (C) Cumulative number of viable T cells 7 days after activation with different APC cells using 0.3 µM of Ova peptide in either polarizing or non-polarizing conditions. Artificial APC lines were treated with mitomycin C for 1 h prior to priming 0.5×106 naïve CD4+ T cells. B7: CHO-IAD-B7; Dll1: CHO-IAD-B7-DLL1; J1: CHO-IAD-B7-Jag1; & Spl: irradiated splenocytes from BALB/c mice. Results are mean±SD from three independent experiments.
(6.95 MB TIF)

Figure S2 Notch ligands cannot instruct Th1/Th2 differentiation but only enhance IFN-γ and IL-4 production with inducing cytokines. (A–C) The flow cytometry plots of three independent experiments presented in Fig. 2A, B. Cells were gated on live CD4+ T cells. Equal numbers of T cells were re-stimulated on Day 7 with PMA/Ionomycin for 4 h in the presence of Brefeldin A. Intracellular cytokines staining were carried out using the following antibodies: APC-conjugated INFγ and PE-conjugated anti-IL-4 antibodies were used for experiment 1; PE-conjugated INFγ and APC-conjugated anti-IL-4 antibodies were used for experiment 2 and 3. Note that IL-4 ICS is highly variable and is dependent on the types of conjugated antibodies used.
(8.26 MB TIF)

Table S1 Proposed roles of Notch signaling in peripheral T cell development. Table 1 summarized the proposed regulatory roles of Notch signaling in T cell activation/proliferation (A) and Th1/Th2 differentiation (B). The references are labeled with either Arabic or Roman numeral to highlight their conflicting conclusions regarding the functions of Notch in these processes.
(0.08 MB DOC)

Table S2 Summary of the level of IFN-γ produced (A) Percent of CD4+ T cells stained positive for intracellular IFN-γ from three independent APC-primed experiments described in Fig. 1 & 2. The ICS values were presented as flow cytometry plot in Supplemental Fig. 2A–C. The mean and standard deviation was calculated using the percent of IFN-γ positive cells and presented graphically in Fig. 2A. (B) The level of IFN-γ secreted by T cells activated with various APCs lines under different polarizing conditions. Equal numbers of T cells were re-stimulated on Day 7 with anti-CD3 for 24 hr. The supernatant was harvested and the level of secreted cytokines was measured with ELISA.
(0.08 MB DOC)

Table S3 Summary of the level of IL-4 produced (A) Percent of CD4+ T cells stained positive for intracellular IL-4 from three independent APC primed experiments described in Fig. 1 & 2. The ICS values were presented as flow cytometry plots in Supplemental Fig. 2A–C. Note that the values of IL-4 ICS are highly variable and are dependent on the types of conjugated antibodies used. (B) The level of IL-4 secreted by T cells activated with various APCs lines under different polarizing conditions. ELISA of IL-4 was carried out as described in Supplemental Table 2B. The mean and standard deviation was calculated and presented graphically in Fig. 2B.
(0.07 MB DOC)

Table S4 Cell expansion regimens used in the experiments (A) CD4+ T cell passage regimen used for experiments described in Figure 1 & 2. (B) CD4+ T cell passage regimen 2 where T cells were expanded at a fixed time schedule regardless of their density in culture. Individual data point was denoted as circle in Figure 3–6. (C) CD4+ T cell passage regimen 3 where T cells were expanded accordingly to their density in culture. Individual data point was denoted as diamond in Figure 3–6. “Seed” stands for the activation of naïve CD4+ T cells with anti-CD3/CD28 antibodies under Th1 or Th2 polarizing conditions. “#w” and “T#” denote the size of the culture flask. For example: 24w denotes 24-well plate and T25 denotes T25 culture flask. “ReST” stands for re-stimulation.
(0.06 MB DOC)
