# Supplementary figures and images for: Notch and Presenilin Regulate Cellular Expansion and Cytokine Secretion but Cannot Instruct Th1/Th2 Fate Acquisition
Source: PLoS One. 2008 Jul 30;3(7):e2823. doi: 10.1371/journal.pone.0002823 (PMC2474705; doi:10.1371/journal.pone.0002823)

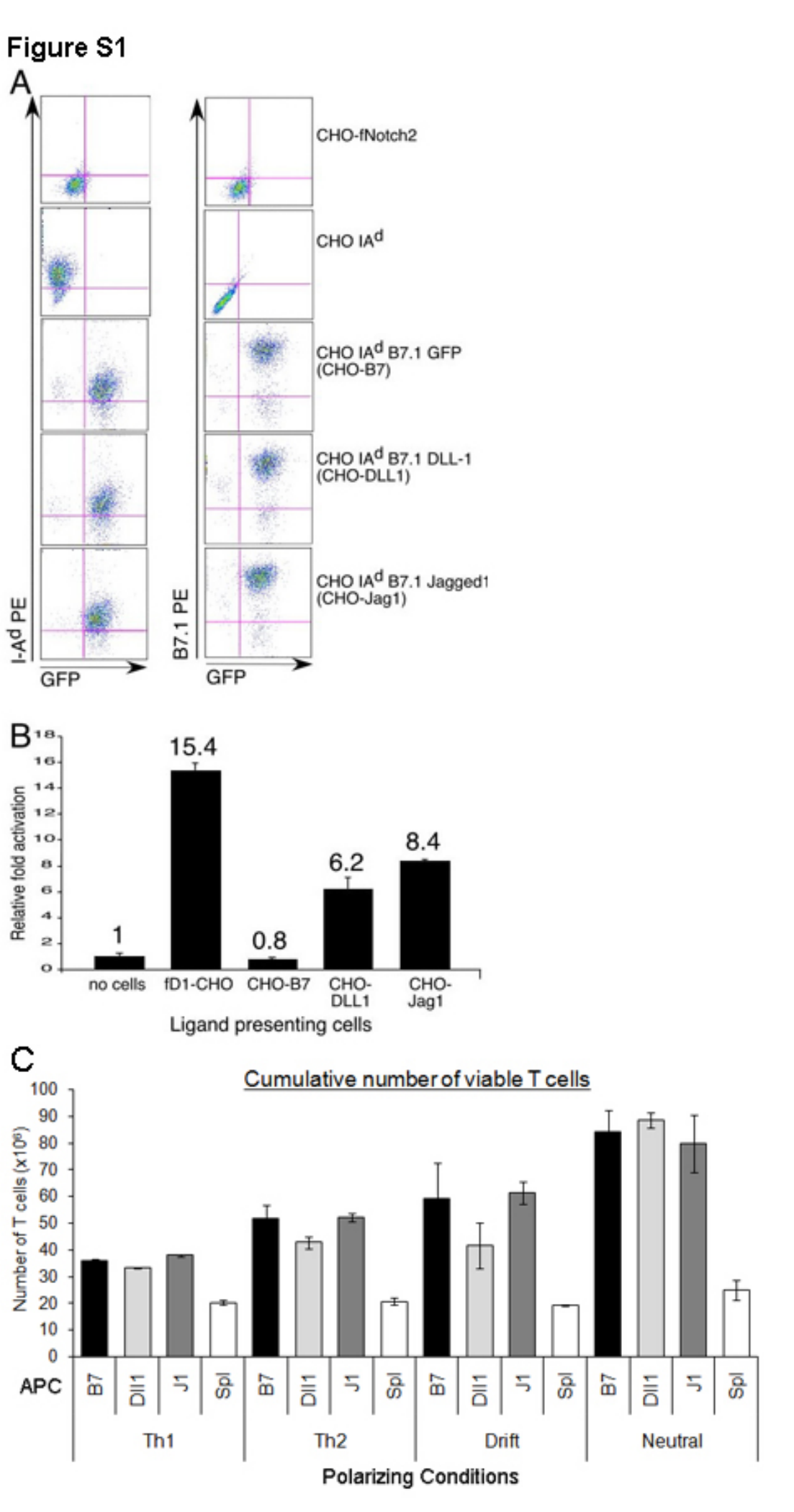

Supplement: Figure S1 — Characterization of the artificial APCs lines that express Notch ligands (A) Expression of I-Ad and B7.1 on CHO cells. CHOfNotch2 is a CHO line that stably expresses full-length Notch2 receptor (Shimizu et al., 2000). CHO-I-Ad expresses I-Ad MHC molecule but not B7.1 ligand. The APC parental line used for the priming experiment contained both I-Ad & B7.1 molecules. CHO-B7 (control line) was generated by infection with empty GFP vector, CHO-DLL1 by infection with DLL1-ires-GFP-RV construct, and CHO-Jag1 with Jagged1-ires-GFP-RV construct. Left: Staining with PE-conjugated I-Ad antibody. Right: Staining with PE-conjugated B7-1 antibody. X-axis is GFP signal. (B) Luciferase assay with co-culture experiments indicates comparable Notch ligand activity in our APCs lines. CHOfNotch2 cells was transfected with TP1-luciferase and PCS2+βgal constructs for 24 h before co-culturing with different ligands expressing cells. The fD1-CHO cell, a published line that exhibits functional ligand activity (Shimizu et al., 2000), was used a positive control in the experiment. (C) Cumulative number of viable T cells 7 days after activation with different APC cells using 0.3 µM of Ova peptide in either polarizing or non-polarizing conditions. Artificial APC lines were treated with mitomycin C for 1 h prior to priming 0.5×10^6^ naïve CD4+ T cells. B7: CHO-IAD-B7; Dll1: CHO-IAD-B7-DLL1; J1: CHO-IAD-B7-Jag1; & Spl: irradiated splenocytes from BALB/c mice. Results are mean±SD from three independent experiments. (6.95 MB TIF) [file pone.0002823.s001.tif]

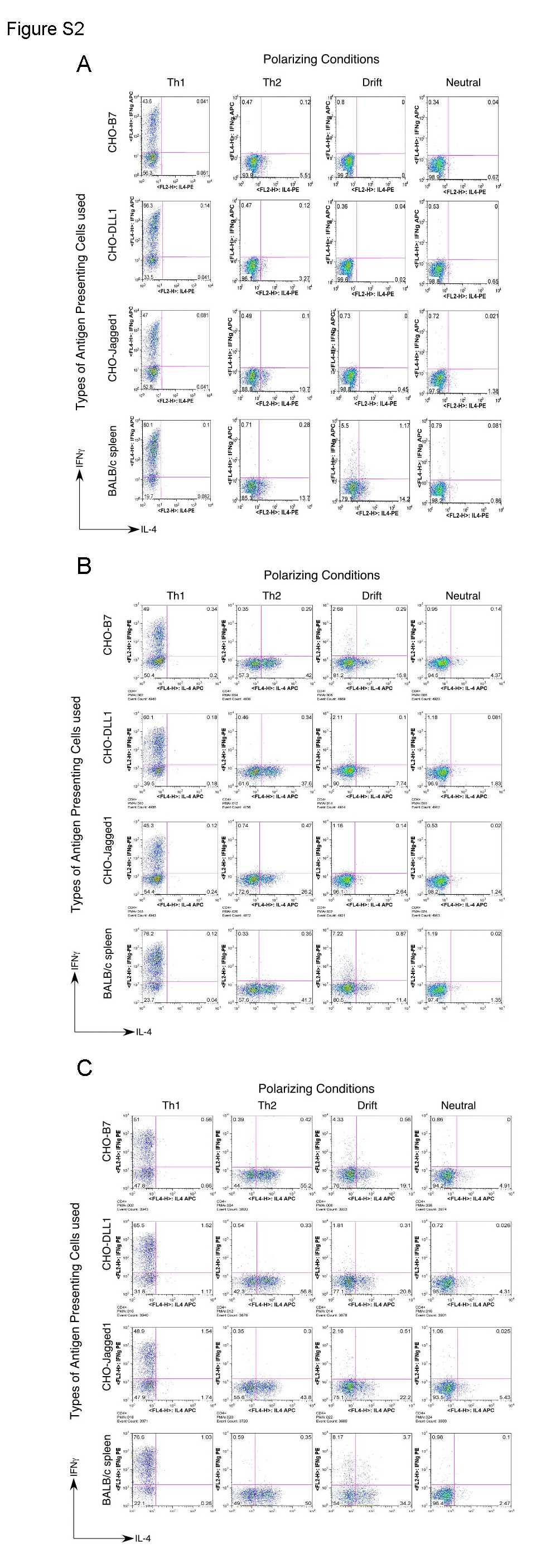

Supplement: Figure S2 — Notch ligands cannot instruct Th1/Th2 differentiation but only enhance IFN-γ and IL-4 production with inducing cytokines. (A–C) The flow cytometry plots of three independent experiments presented in Fig. 2A, B. Cells were gated on live CD4+ T cells. Equal numbers of T cells were re-stimulated on Day 7 with PMA/Ionomycin for 4 h in the presence of Brefeldin A. Intracellular cytokines staining were carried out using the following antibodies: APC-conjugated INFγ and PE-conjugated anti-IL-4 antibodies were used for experiment 1; PE-conjugated INFγ and APC-conjugated anti-IL-4 antibodies were used for experiment 2 and 3. Note that IL-4 ICS is highly variable and is dependent on the types of conjugated antibodies used. (8.26 MB TIF) [file pone.0002823.s002.tif]
