# Supplementary material for: Notch and Presenilin Regulate Cellular Expansion and Cytokine Secretion but Cannot Instruct Th1/Th2 Fate Acquisition
Source: PLoS One. 2008 Jul 30;3(7):e2823. doi: 10.1371/journal.pone.0002823 (PMC2474705; doi:10.1371/journal.pone.0002823)
Supplement: Table S1 — Proposed roles of Notch signaling in peripheral T cell development. Table 1 summarized the proposed regulatory roles of Notch signaling in T cell activation/proliferation (A) and Th1/Th2 differentiation (B). The references are labeled with either Arabic or Roman numeral to highlight their conflicting conclusions regarding the functions of Notch in these processes. (0.08 MB DOC) [file pone.0002823.s003.doc]

**Supplemental Table 1A:** The proposed roles of Notch signaling in regulating T cell activation, proliferation and survival.

| **T cell activation, proliferation and survival** | | |
| --- | --- | --- |
| **#** | Reference & Major Approaches | **Key Conclusions** |
| 1 | (Palaga et al., 2003).  GSI treatment of T cell activated by anti-CD3/CD28 or Con A. | Notch up-regulates T cell proliferation & IFN-γ production. |
| 2 | (Adler et al., 2003).  GSI treatment of DO11.10 T cells activated by APCs and over-expression of NICD1 in activated T cells. | Notch augments T cell responsiveness through CD25. |
| 3 | (Tanigaki et al., 2004).  Activation of RBP-J-deficient T cells with antiCD3/CD28 in the presence or absence of APCs. | Reduced proliferation of RBP-J-deficient cells in the presence of APC.  RBP-J-deficient cells have preferential Th1 response. |
| 4 | (Bheeshmachar et al., 2006).  Addition of GSI or Notch1-blocking antibody to activated T cells and over-expression of NICD1 in activated T cells. | Notch1 signaling positively regulates cytokine-dependent survival of activated T cells. |
| **i** | (Eagar et al., 2004).  Activation of T cells with anti-CD3/CD28 in the presence or absence of anti-Notch1 antibody, Jag1 or DLL1 beads. | Notch negatively regulates T cell activation & proliferation in an γ-secretase dependent manner. |
| **ii** | (Benson et al., 2005).  GSI (MW167 or JLK6) treatment of activated T cells and over-expression of NICD1 in naïve human CD4+ T cells. | JLK6 but not MW167 blocks T cell proliferation. In contrast, MW167 but not JLK6 blocks IL-10 production. Overexpression of NICD1 inhibits T cell proliferation but promotes the secretion of IL-4, IL-10, IFN-γ. |
| **iii** | (Rutz et al., 2005).  Activation of T cells with anti-CD3/CD28 in the presence of DLL1, DLL4 or Jag1 fusion protein, with GSI (DAPT) treatment in some experiments. | DLL1 and Jag1 inhibit early T cell activation markers and proliferation while DLL4 enhances these processes, an effect that is independent of γ-secretase activity. |

**Supplemental Table 1B:** The proposed roles of Notch signaling in regulating Th1 and Th2 differentiation.

| **Th1 and Th2 differentiation** | | |
| --- | --- | --- |
| **#** | Author & Major Approaches | **Key Conclusions** |
| 1 | (Maekawa et al., 2003).  Activation of T cells with DLL1-Fc and anti-CD3. Antisense treatment of Notch3 in T cells. *L. major* infected BALB/C mice were treated with DLL1-Fc. | DLL1-Notch3 interaction bias Th1 fate by up-regulating T-bet expression. |
| 2 | (Amsen et al., 2004).  AND T cells were primed with DCEK hi7 cells presenting Jag1 or DLL1 cells.  RBP-J-deficient cells were primed with LPS pretreated BMDCs. Over-expression of NICD1 in activated T cells. | DLL1 instructs Th1 fate while Jag1 instructs Th2 fate by NICD1 up-regulation of IL-4 expression. |
| 3 | (Tu et al., 2005).  *In vitro* polarization, *L.major* or *T. muris* challenge of mice that over-express DN-MAML in CD4^+^ T cells. | Canonical Notch pathway is important regulator of Th2 but not Th1 immunity. |
| 4 | (Amsen et al., 2007).  RBP-J-deficient or Notch1/2-deficient T cells were primed with SEA pretreated APC. ChIP and EMSA of GATA-3 regulatory elements. Over-expression of NICD1 in WT or GATA-3 null T cells. | Direct regulation of GATA-3 by Notch determines its Th2 differentiation potential. |
| 5 | (Fang et al., 2007).  WT, GSI treated or DN-MAML T cells were activated with APCs. ChIP and EMSA of GATA-3 regulatory elements. Over-expression of NICD in the presence or absence of DN-GATA-3 in T cells. | Notch directly regulates GATA-3 expression during Th2 differentiation. |
| **i** | (Tacchini-Cottier et al., 2004).  *In vitro* polarization and *L. major* infection of Notch1-deficient T cells. | Notch1 is not required for Th1/Th2 differentiation. |
| **ii** | (Minter et al., 2005).  GSI treatment of *in vitro* activated T cells or mice with EAE. ChIP of T-bet promoter in DO11.10 T cell hybidomas that express NICD1 stably. | GSI blocks Th1 development by preventing Notch1 activation of T-bet. |

## References S1

## Adler, S. H., Chiffoleau, E., Xu, L., Dalton, N. M., Burg, J. M., Wells, A. D., Wolfe, M. S., Turka, L. A., and Pear, W. S. (2003). Notch signaling augments T cell responsiveness by enhancing CD25 expression. J Immunol *171*, 2896-2903.

## Amsen, D., Antov, A., Jankovic, D., Sher, A., Radtke, F., Souabni, A., Busslinger, M., McCright, B., Gridley, T., and Flavell, R. A. (2007). Direct regulation of gata3 expression determines the T helper differentiation potential of notch. Immunity *27*, 89-99.

## Amsen, D., Blander, J. M., Lee, G. R., Tanigaki, K., Honjo, T., and Flavell, R. A. (2004). Instruction of distinct CD4 T helper cell fates by different notch ligands on antigen-presenting cells. Cell *117*, 515-526.

## Benson, R. A., Adamson, K., Corsin-Jimenez, M., Marley, J. V., Wahl, K. A., Lamb, J. R., and Howie, S. E. (2005). Notch1 co-localizes with CD4 on activated T cells and Notch signaling is required for IL-10 production. Eur J Immunol *35*, 859-869.

## Bheeshmachar, G., Purushotaman, D., Sade, H., Gunasekharan, V., Rangarajan, A., and Sarin, A. (2006). Evidence for a role for notch signaling in the cytokine-dependent survival of activated T cells. J Immunol *177*, 5041-5050.

## Eagar, T. N., Tang, Q., Wolfe, M., He, Y., Pear, W. S., and Bluestone, J. A. (2004). Notch 1 signaling regulates peripheral T cell activation. Immunity *20*, 407-415.

## Fang, T. C., Yashiro-Ohtani, Y., Del Bianco, C., Knoblock, D. M., Blacklow, S. C., and Pear, W. S. (2007). Notch directly regulates Gata3 expression during T helper 2 cell differentiation. Immunity *27*, 100-110.

## Maekawa, Y., Tsukumo, S., Chiba, S., Hirai, H., Hayashi, Y., Okada, H., Kishihara, K., and Yasutomo, K. (2003). Delta1-Notch3 interactions bias the functional differentiation of activated CD4+ T cells. Immunity *19*, 549-559.

## Minter, L. M., Turley, D. M., Das, P., Shin, H. M., Joshi, I., Lawlor, R. G., Cho, O. H., Palaga, T., Gottipati, S., Telfer, J. C.*, et al.* (2005). Inhibitors of gamma-secretase block in vivo and in vitro T helper type 1 polarization by preventing Notch upregulation of Tbx21. Nat Immunol *6*, 680-688.

## Palaga, T., Miele, L., Golde, T. E., and Osborne, B. A. (2003). TCR-mediated Notch signaling regulates proliferation and IFN-gamma production in peripheral T cells. J Immunol *171*, 3019-3024.

## Rutz, S., Mordmuller, B., Sakano, S., and Scheffold, A. (2005). Notch ligands Delta-like1, Delta-like4 and Jagged1 differentially regulate activation of peripheral T helper cells. Eur J Immunol *35*, 2443-2451.

## Shimizu, K., Chiba, S., Hosoya, N., Kumano, K., Saito, T., Kurokawa, M., Kanda, Y., Hamada, Y., and Hirai, H. (2000). Binding of Delta1, Jagged1, and Jagged2 to Notch2 rapidly induces cleavage, nuclear translocation, and hyperphosphorylation of Notch2. Mol Cell Biol *20*, 6913-6922.

## Tacchini-Cottier, F., Allenbach, C., Otten, L. A., and Radtke, F. (2004). Notch1 expression on T cells is not required for CD4+ T helper differentiation. Eur J Immunol *34*, 1588-1596.

## Tanigaki, K., Tsuji, M., Yamamoto, N., Han, H., Tsukada, J., Inoue, H., Kubo, M., and Honjo, T. (2004). Regulation of alphabeta/gammadelta T cell lineage commitment and peripheral T cell responses by Notch/RBP-J signaling. Immunity *20*, 611-622.

## Tu, L., Fang, T. C., Artis, D., Shestova, O., Pross, S. E., Maillard, I., and Pear, W. S. (2005). Notch signaling is an important regulator of type 2 immunity. J Exp Med *202*, 1037-1042.
