# Supplementary material for: Notch and Presenilin Regulate Cellular Expansion and Cytokine Secretion but Cannot Instruct Th1/Th2 Fate Acquisition
Source: PLoS One. 2008 Jul 30;3(7):e2823. doi: 10.1371/journal.pone.0002823 (PMC2474705; doi:10.1371/journal.pone.0002823)
Supplement: Table S2 — Summary of the level of IFN-γ produced (A) Percent of CD4+ T cells stained positive for intracellular IFN-γ from three independent APC-primed experiments described in Fig. 1 & 2. The ICS values were presented as flow cytometry plot in Supplemental Fig. 2A–C. The mean and standard deviation was calculated using the percent of IFN-γ positive cells and presented graphically in Fig. 2A. (B) The level of IFN-γ secreted by T cells activated with various APCs lines under different polarizing conditions. Equal numbers of T cells were re-stimulated on Day 7 with anti-CD3 for 24 hr. The supernatant was harvested and the level of secreted cytokines was measured with ELISA. (0.08 MB DOC) [file pone.0002823.s004.doc]

**Supplemental Table 2A** Percent of IFN-γ positive CD4 +T cells from three independent experiments

| **Polarizing Conditions** | **Types of APCs** | **% of IFN-γ positive cells** | | | **Mean** | **SD** |
| --- | --- | --- | --- | --- | --- | --- |
|  |  | **Expt1** | **Expt2** | **Expt3** |  |  |
| Th1 | CHO-B7 | 43.60 | 49.00 | 51 | 47.87 | 3.83 |
|  | CHO-DL1 | 66.30 | 60.10 | 65.5 | 63.97 | 3.37 |
|  | CHO-J1 | 47.00 | 45.30 | 48.9 | 47.07 | 1.80 |
|  | Balb/c spl | 80.10 | 76.20 | 76.6 | 77.63 | 2.15 |
| Th2 | CHO-B7 | 0.47 | 0.35 | 0.39 | 0.40 | 0.06 |
|  | CHO-DL1 | 0.47 | 0.46 | 0.54 | 0.49 | 0.04 |
|  | CHO-J1 | 0.49 | 0.74 | 0.35 | 0.53 | 0.20 |
|  | Balb/c spl | 0.71 | 0.33 | 0.59 | 0.54 | 0.19 |
| Drift | CHO-B7 | 0.80 | 2.68 | 4.33 | 2.60 | 1.77 |
|  | CHO-DL1 | 0.36 | 2.11 | 1.81 | 1.43 | 0.94 |
|  | CHO-J1 | 0.73 | 1.16 | 2.16 | 1.35 | 0.73 |
|  | Balb/c spl | 5.50 | 7.22 | 8.17 | 6.96 | 1.35 |
| Neutral | CHO-B7 | 0.34 | 0.95 | 0.86 | 0.72 | 0.33 |
|  | CHO-DL1 | 0.53 | 1.18 | 0.72 | 0.81 | 0.33 |
|  | CHO-J1 | 0.72 | 0.53 | 1.06 | 0.77 | 0.27 |
|  | Balb/c spl | 0.79 | 1.19 | 0.98 | 0.99 | 0.20 |

### **Supplemental Table 2B** Level of secreted IFN-γ from three independent experiments

| **Polarizing Conditions** | **Types of APCs** | **Level of secreted IFN-γ (pg/ml)** | | | **Mean** | **SD** |
| --- | --- | --- | --- | --- | --- | --- |
|  |  | **Expt1** | **Expt2** | **Expt3** |  |  |
| Th1 | CHO-B7 | 8535 | 7978 | 2144 | 6219 | 3540 |
|  | CHO-DL1 | 15352 | 9799 | 3467 | 9539 | 5947 |
|  | CHO-J1 | 8287 | 5979 | 3499 | 5922 | 2395 |
|  | Balb/c spl | 13016 | 15936 | 7408 | 12120 | 4334 |
| Th2 | CHO-B7 | 455 | 179 | 200 | 278 | 154 |
|  | CHO-DL1 | 511 | 264 | 191 | 322 | 168 |
|  | CHO-J1 | 444 | 177 | 168 | 263 | 157 |
|  | Balb/c spl | 341 | 159 | 360 | 287 | 111 |
| Drift | CHO-B7 | 567 | 506 | 425 | 499 | 71 |
|  | CHO-DL1 | 1110 | 1082 | 490 | 894 | 350 |
|  | CHO-J1 | 644 | 345 | 494 | 494 | 149 |
|  | Balb/c spl | 973 | 1418 | 1267 | 1220 | 226 |
| Neutral | CHO-B7 | 507 | 199 | 114 | 273 | 207 |
|  | CHO-DL1 | 988 | 204 | 162 | 451 | 465 |
|  | CHO-J1 | 586 | 109 | 160 | 285 | 262 |
|  | Balb/c spl | 549 | 310 | 218 | 359 | 171 |
