# Supplementary material for: Notch and Presenilin Regulate Cellular Expansion and Cytokine Secretion but Cannot Instruct Th1/Th2 Fate Acquisition
Source: PLoS One. 2008 Jul 30;3(7):e2823. doi: 10.1371/journal.pone.0002823 (PMC2474705; doi:10.1371/journal.pone.0002823)
Supplement: Table S3 — Summary of the level of IL-4 produced (A) Percent of CD4+ T cells stained positive for intracellular IL-4 from three independent APC primed experiments described in Fig. 1 & 2. The ICS values were presented as flow cytometry plots in Supplemental Fig. 2A–C. Note that the values of IL-4 ICS are highly variable and are dependent on the types of conjugated antibodies used. (B) The level of IL-4 secreted by T cells activated with various APCs lines under different polarizing conditions. ELISA of IL-4 was carried out as described in Supplemental Table 2B. The mean and standard deviation was calculated and presented graphically in Fig. 2B. (0.07 MB DOC) [file pone.0002823.s005.doc]

Supplemental Table 3A Percent of IL-4 positive CD4+ T cells from three independent experiments

| **Polarizing Conditions** | **Types of APC** | **% of IL-4 positive cells** | | |
| --- | --- | --- | --- | --- |
|  |  | **IL4-PE** | **IL4-APC** | |
|  |  | **Expt1** | **Expt2** | **Expt3** |
| Th1 | CHO-B7 | 0.06 | 0.20 | 0.66 |
|  | CHO-DL1 | 0.04 | 0.18 | 1.17 |
|  | CHO-J1 | 0.04 | 0.24 | 1.74 |
|  | Balb/c spl | 0.08 | 0.04 | 0.26 |
| Th2 | CHO-B7 | 5.51 | 42.00 | 55.2 |
|  | CHO-DL1 | 3.27 | 37.60 | 56.8 |
|  | CHO-J1 | 10.70 | 26.20 | 43.8 |
|  | Balb/c spl | 13.70 | 41.70 | 50 |
| Drift | CHO-B7 | 0.00 | 15.80 | 19.1 |
|  | CHO-DL1 | 0.02 | 7.74 | 20.8 |
|  | CHO-J1 | 0.45 | 2.64 | 22.2 |
|  | Balb/c spl | 14.20 | 11.40 | 34.2 |
| Neutral | CHO-B7 | 0.67 | 4.37 | 4.91 |
|  | CHO-DL1 | 0.65 | 1.83 | 4.31 |
|  | CHO-J1 | 1.38 | 1.24 | 5.43 |
|  | Balb/c spl | 0.86 | 1.35 | 2.47 |

Supplemental Table 3B Level of secreted IL-4 from three independent experiments

| **Polarizing Conditions** | **Types of APC** | **Level of secreted IL-4 (pg/ml)** | | | **Mean** | **SD** |
| --- | --- | --- | --- | --- | --- | --- |
|  |  | **Expt1** | **Expt2** | **Expt3** |  |  |
| Th1 | CHO-B7 | 115 | 81 | 60 | 85 | 28 |
|  | CHO-DL1 | 110 | 88 | 69 | 89 | 20 |
|  | CHO-J1 | 120 | 77 | 127 | 108 | 27 |
|  | Balb/c spl | 144 | 77 | 60 | 94 | 44 |
| Th2 | CHO-B7 | 2497 | 3304 | 2226 | 2676 | 560 |
|  | CHO-DL1 | 5294 | 4383 | 4381 | 4686 | 527 |
|  | CHO-J1 | 3614 | 3411 | 4143 | 3722 | 378 |
|  | Balb/c spl | 1488 | 2787 | 2181 | 2152 | 650 |
| Drift | CHO-B7 | 1283 | 683 | 499 | 822 | 410 |
|  | CHO-DL1 | 1459 | 907 | 751 | 1039 | 372 |
|  | CHO-J1 | 1279 | 621 | 926 | 942 | 329 |
|  | Balb/c spl | 1623 | 741 | 1724 | 1363 | 541 |
| Neutral | CHO-B7 | 480 | 354 | 123 | 319 | 181 |
|  | CHO-DL1 | 875 | 380 | 135 | 463 | 377 |
|  | CHO-J1 | 543 | 327 | 182 | 351 | 182 |
|  | Balb/c spl | 157 | 135 | 79 | 124 | 40 |
