# Supplementary material for: Notch and Presenilin Regulate Cellular Expansion and Cytokine Secretion but Cannot Instruct Th1/Th2 Fate Acquisition
Source: PLoS One. 2008 Jul 30;3(7):e2823. doi: 10.1371/journal.pone.0002823 (PMC2474705; doi:10.1371/journal.pone.0002823)
Supplement: Table S4 — Cell expansion regimens used in the experiments (A) CD4+ T cell passage regimen used for experiments described in Figure 1 & 2. (B) CD4+ T cell passage regimen 2 where T cells were expanded at a fixed time schedule regardless of their density in culture. Individual data point was denoted as circle in Figure 3– 6. (C) CD4+ T cell passage regimen 3 where T cells were expanded accordingly to their density in culture. Individual data point was denoted as diamond in Figure 3– 6. “Seed” stands for the activation of naïve CD4+ T cells with anti-CD3/CD28 antibodies under Th1 or Th2 polarizing conditions. “#w” and “T#” denote the size of the culture flask. For example: 24w denotes 24-well plate and T25 denotes T25 culture flask. “ReST” stands for re-stimulation. (0.06 MB DOC) [file pone.0002823.s006.doc]

**Supplemental Table 4A** Passage Regimen One

| Day | **Culture** | **Procedure** |
| --- | --- | --- |
| 0 | 48 well | Purification of naïve CD4+ T cells from DO11.10 mice (>98%).  Primed with 0.3 μM OVA peptide and different APCs lines in Th1, Th2, Drift or Neutral conditions. |
| 1 | 48 well |  |
| 2 | 4x48 well | Split into new medium containing IL-2 cytokine. |
| 3 | T25 | Split into new medium containing IL-2 cytokine. |
| 4 | T165 | Split into new medium containing IL-2 cytokine. |
| 5 | 2xT165 | Split into new medium containing IL-2 cytokine. |
| 6 | 2xT165 & 1xT75 | Split into new medium containing IL-2 cytokine. |
| 7 | 48 well | Count cells.  Re-stimulation of 0.5x10^6^ cells with anti-CD3 antibody (24 hr) for ELISA.  PMA/Ionomycin in the presence of BFA (4hr) for intracellular staining of cytokines. |
| 8 |  | ELISA of the supernatant. |

**Supplemental Table 4B** Passage Regimen Two

| Day | **Culture** | **Procedure** |
| --- | --- | --- |
| 0 | 48 well | Purification of naïve CD4+ T cells from different genotypes (>95%).  Anti-CD3/anti-CD28 stimulation in either Th1 or Th2 condition. |
| 1 | 48 well |  |
| 2 | 12 well | Split ALL genotypes into new medium containing IL-2 cytokine. |
| 3 | T25 | Split ALL genotypes into new medium containing IL-2 cytokine. |
| 4 | T75/T165 | Split ALL genotypes into new medium containing IL-2 cytokine. |
| 5 |  |  |
| 6 | 48 well | Count cells.  Re-stimulation of 0.5x10^6^ cells with anti-CD3 antibody or PMA/Ionomycin. |
| 7 |  | Add BFA for the final four hours of re-stimulation.  Assay by intracellular staining for cytokines and ELISA of the supernatant. |

## Supplemental Table 4C Passage Regimen Three

##### Passage regimen for “diamond” data points in Figures 3, 4, 5 & 6

|  | **D0** | **D1** | **D2** | **D3** | **D4** | **D5** | D6 | **D7** | **Th1** | **Th2** |
| --- | --- | --- | --- | --- | --- | --- | --- | --- | --- | --- |
| Expt R1 | | | | | | | | | | |
| PSdko | Seed |  | 24w | 12w | T25 |  | ReST |  |  |  |
| *Het* | Seed |  | 24w | T25 | T75 |  | ReST |  |  |  |
| Expt R2 | | | | | | | | | | |
| Het | Seed |  | 12w | T25 | T75+T25 | T165 | ReST |  |  |  |
| *PSdko* | Seed |  | 24w | 12w | 6w | Add media | ReST |  |  |  |
| *PSdko* | Seed |  | 12w | T25 | T25+T25 | T75 | ReST |  |  |  |
| *PSRtko* | Seed |  | 12w | T25 | T75+T25 | T165 | ReST |  |  |  |
| *PSRtko* | Seed |  | 12w | T25 | T25+T25 | T75 | ReST |  |  |  |
